# Supplementary material for: Ridge-assisted Micro Positioning of Cells and Particles in a Microchannel
Source: bioRxiv. 2025 Sep 29:2025.09.26.678893. Preprint. [Version 1] doi: 10.1101/2025.09.26.678893 (PMC12621959; doi:10.1101/2025.09.26.678893)
Supplement: Supplement 1 [file media-1.pdf]

Supplementary Materials for

Ridge-Assisted Micro Positioning of Cells and Particles in a Microchannel

Adriana Payan-Medina *et al.*

*Corresponding author. Email: adripm@mit.edu

This PDF file includes:

Supplementary Text
Figs. S1 to S8
Tables S1 to S6
Movies S1 to S8
References (1 to 13)

Other Supplementary Materials for this manuscript include the following:
Movies S1 to S8

Supplementary Text

Chevron Theory for Inertial Focusing

Trapping in ridge-assisted microfluidic devices is governed by a balance of secondary-flow-induced drag and lift forces. Since trapping is occurring away from the top and side walls, we can approximate the lift force as $F_L = C_L \rho U^2 a^4/H^2$, where C_L is the lift coefficient, ρ is the fluid density, U is the average velocity, a is the particle diameter, and H is the height of the channel. Theoretical treatment of secondary flow induced by ridges has been presented in (*1*). Following their analysis for thin channels ($W >> H$) and shallow grooves ($h/H << 1$), the drag force on a particle can be written as $F_D = 3\pi\mu a U_{y1}$. Thus, the ratio of lift to drag force can be written as **Equation S1**.

Equation S1.

$$\frac{F_L}{F_D} = \frac{\frac{C_L \rho U^2 a^4}{H^2}}{3\pi\mu a U_{y1}}$$

Equation S2.

$$U_{y1} = 6\alpha^2 U \left(\frac{3}{2}\frac{(H-z)(z)}{H^2} - \frac{z}{2H}\right)\left(\frac{2\pi H}{\lambda} - 1\right) \mathrm{Cos}\,\theta$$

In **Equation S2,** λ is the wavelength, θ is the angle of the ridges, and α is a geometrical nondimensional number. A relation for ridge height (h) is presented as $h = 2\alpha H$. Thus, the lift-to-drag force ratio can be approximated as given in **Equation S3**.

Equation S3.

$$\frac{F_L}{F_D} = \frac{C_L \rho U}{18\pi\mu\alpha^2 \left(\frac{3}{2}\frac{(H-z)(z)}{H^2} - \frac{z}{2H}\right)\left(\frac{2\pi H}{\lambda} - 1\right) \mathrm{Cos}\,\theta}\, a^3/H^2$$

Assuming that trapping is often seen at $z \cong \frac{3H}{4}$, this equation can be reduced to a more simplified form (**Equation S4**).

Equation S4.

$$\frac{F_L}{F_D} \cong \frac{8}{27\pi^2}\frac{C_L \rho U \lambda}{\mu\alpha^2 \mathrm{Cos}\,\theta}\left(\frac{a}{H}\right)^3$$

Though these results are approximate solutions derived through various assumptions, and the slip velocity will be higher than the vertical component of velocity, these results show that among a few parameters, particle trapping will be strongly affected by confinement (a/H) and α. Based on this analysis, we designed devices with low α and high confinement ratios ($a/H > 0.2$). In this analysis, $C_L = 0.12$, based on the work done in (*2*).

Elastic lift force: Elastic lift force is proportional to normal stress (N_1) variation over the particle volume (a^3). C_e represents the elastic lift coefficient.

Equation S5.

$$F_e = C_e a^3 \nabla N_1$$

Deborah's number (De): De is a ratio of the characteristic time of the fluid (τ) and the characteristic time of the deformation process (t_f).

Equation S6.

$$De = \frac{\tau}{t_f} = \frac{\tau Q}{H^3}$$

Reynolds number (Re): Re is used to compare fluid inertial and viscous forces. Fluid density, average velocity, channel hydraulic diameter, and viscosity are ρ, U, D_h, and μ, respectively.

Equation S7.

$$Re = \frac{\rho U D_h}{\mu}$$

Elasticity number (El): El is a ratio of Deborah's number divided by Reynolds number and can be used to compare a fluid's elastic and inertial properties.

Equation S8.

$$El = \frac{De}{Re}$$

The ratio of elastic lift force and drag force can be written as Equation S9, denoting a weaker dependence on particle size ($\propto a^2$) than the ratio of inertial lift force and drag force ($\propto a^3$).

Equation S9.

$$\frac{F_e}{F_D} = \frac{C_e a^3 \nabla N_1}{3\pi\mu a U_{y1}} = \frac{C_e a^2 \nabla N_1}{3\pi\mu U_{y1}}$$

<u>Computation Modelling</u>

Steady state 3D fluid flow simulations were conducted in COMSOL Multiphysics using the Navier–Stokes equation. A schematic diagram of the boundary conditions is shown in Figure S1, below. A velocity inlet boundary condition was applied at the inlet, an atmospheric pressure outlet boundary condition was applied, and a no-slip boundary condition was imposed on the rest of the surfaces. A free tetrahedral mesh with a minimum element size of 0.5 µm was used. We conducted a mesh refinement study to ensure that the results were independent of mesh element size.

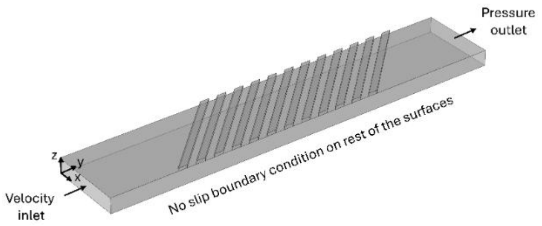

Figure S1. Boundary conditions used in the fluid flow simulation.

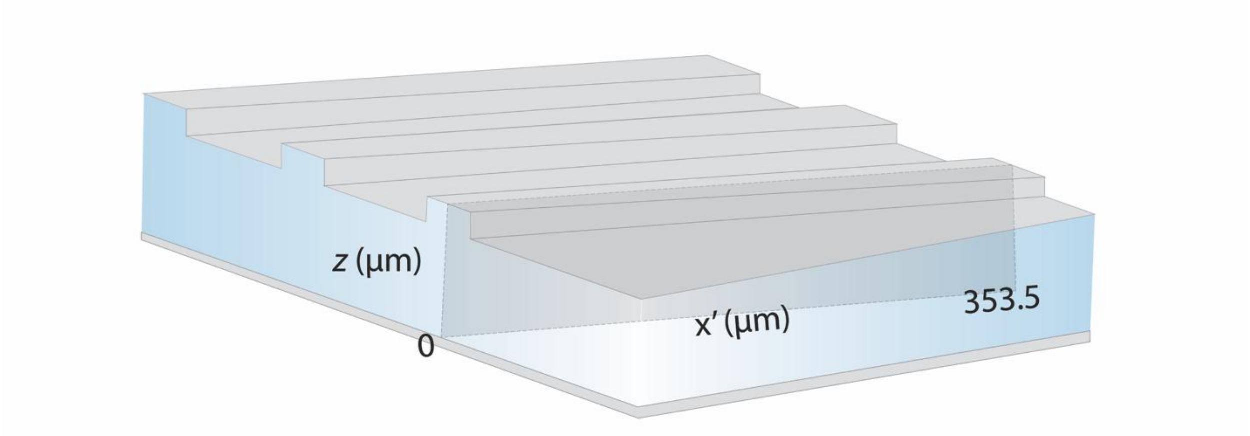

Figure S2. A schematic representation of a RAMP device shows the cross-section parallel to the ridge microstructures used for presenting the velocity field and the microvortex.

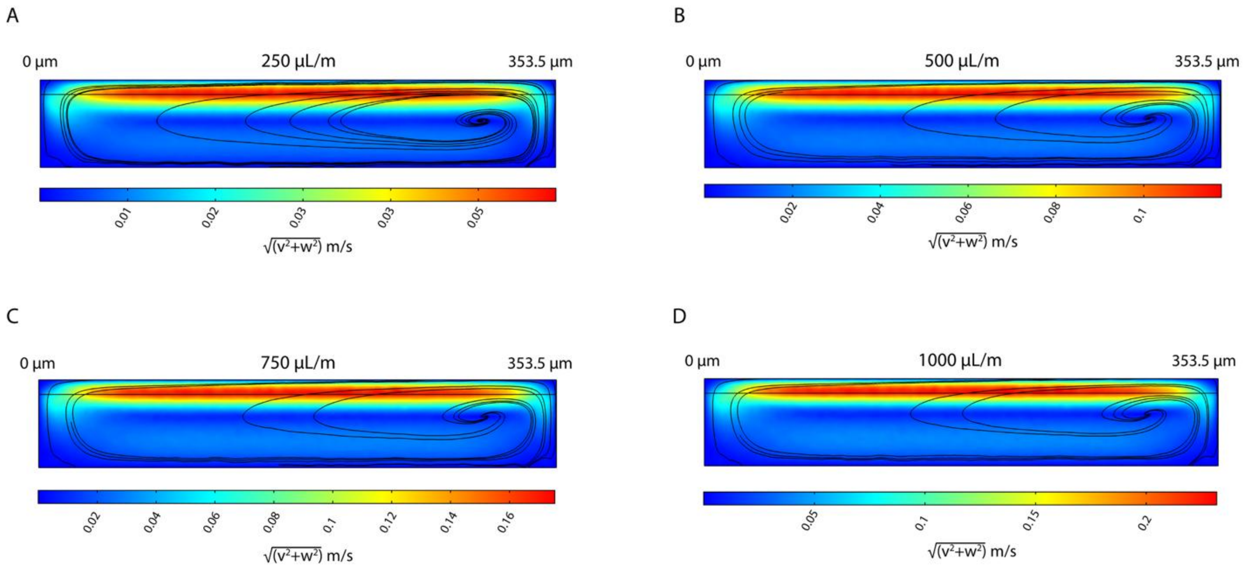

Figure S3. Finite element simulations of fluid flow in a RAMP device show equivalent recirculation regions formed at several flow rates. Simulations were performed at flow rates of (**A**) 250 µL/min, (**B**) 500 µL/min, (**C**) 750 µL/min, and (**D**) 1000 µL/min.

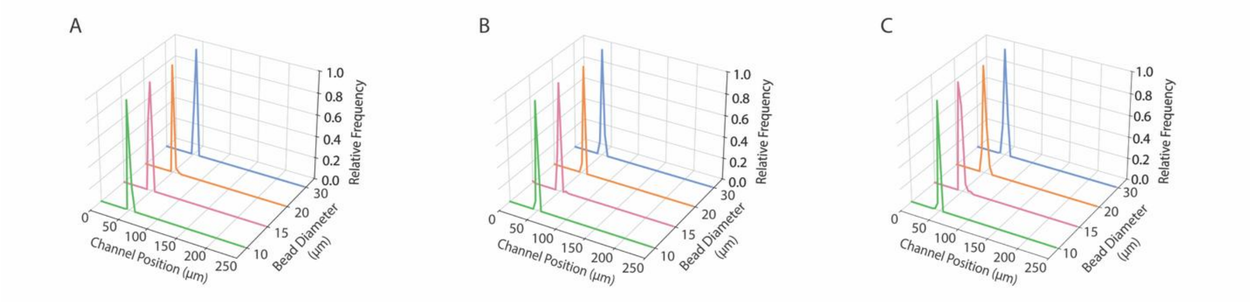

Figure S4. RAMP aligns 10, 15, 20, and 30 µm particles across a wide range of flow rates. Histograms of particle centroid positions are shown for flow rates of (**A**) 500 µL/min, *Re* = 50.4 (n=118 (10 µm), 99 (15 µm), 146 (20 µm), 105 (30 µm) particles), (**B**) 750 µL/min, *Re* = 75.6 (n=180 (10 µm), 99 (15 µm), 71 (20 µm), 143 (30 µm) particles), and (**C**) 1000 µL/min, *Re* = 100.8 (n=132 (10 µm), 99 (15 µm), 165 (20 µm), 163 (30 µm) particles).

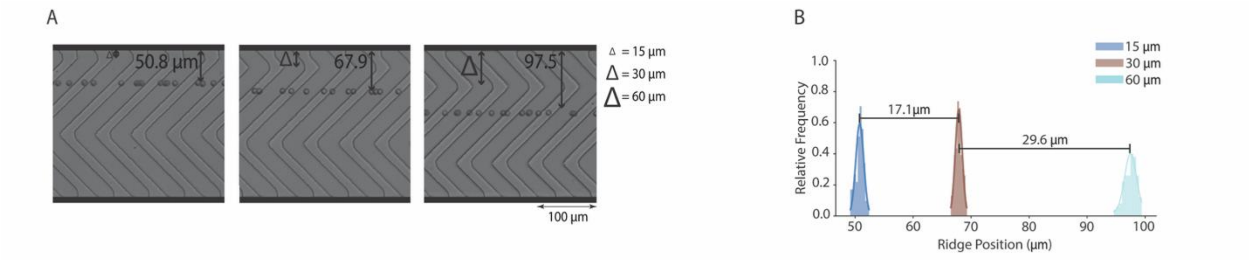

Figure S5. Large focusing position shifts in 10 µm particle streamlines are achieved with proportional shifts in the position of center ridges. (**A**) Shifts in particle focus position are observed with high-speed camera streak images as the central ridge structure is shifted in steps of 15 µm and 30 µm. (**B**) A frequency distribution plot of particle centroid positions shows the lateral streamline shift that occurs as the center ridge is shifted (15 µm (n = 131), 30 µm (n = 83), and 60 µm (n = 105)).

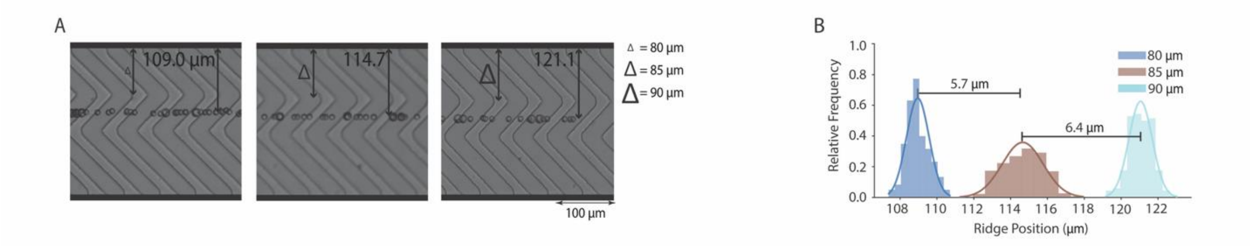

Figure S6. Focusing position shifts in a mixture of 10 µm and 15 µm particles are achieved with proportional shifts in the position of center ridges. (**A**) Shifts in particle focus position are observed with high-speed camera streak images as the position of the central ridge structure is shifted in steps of 5 µm from the channel side wall. (**B**) A frequency distribution plot of particle centroid positions describes the lateral streamline shift that occurs as the center ridge is shifted 80 µm (n = 384), 85 µm (n = 403), and 90 µm (n = 191) from the channel sidewall.

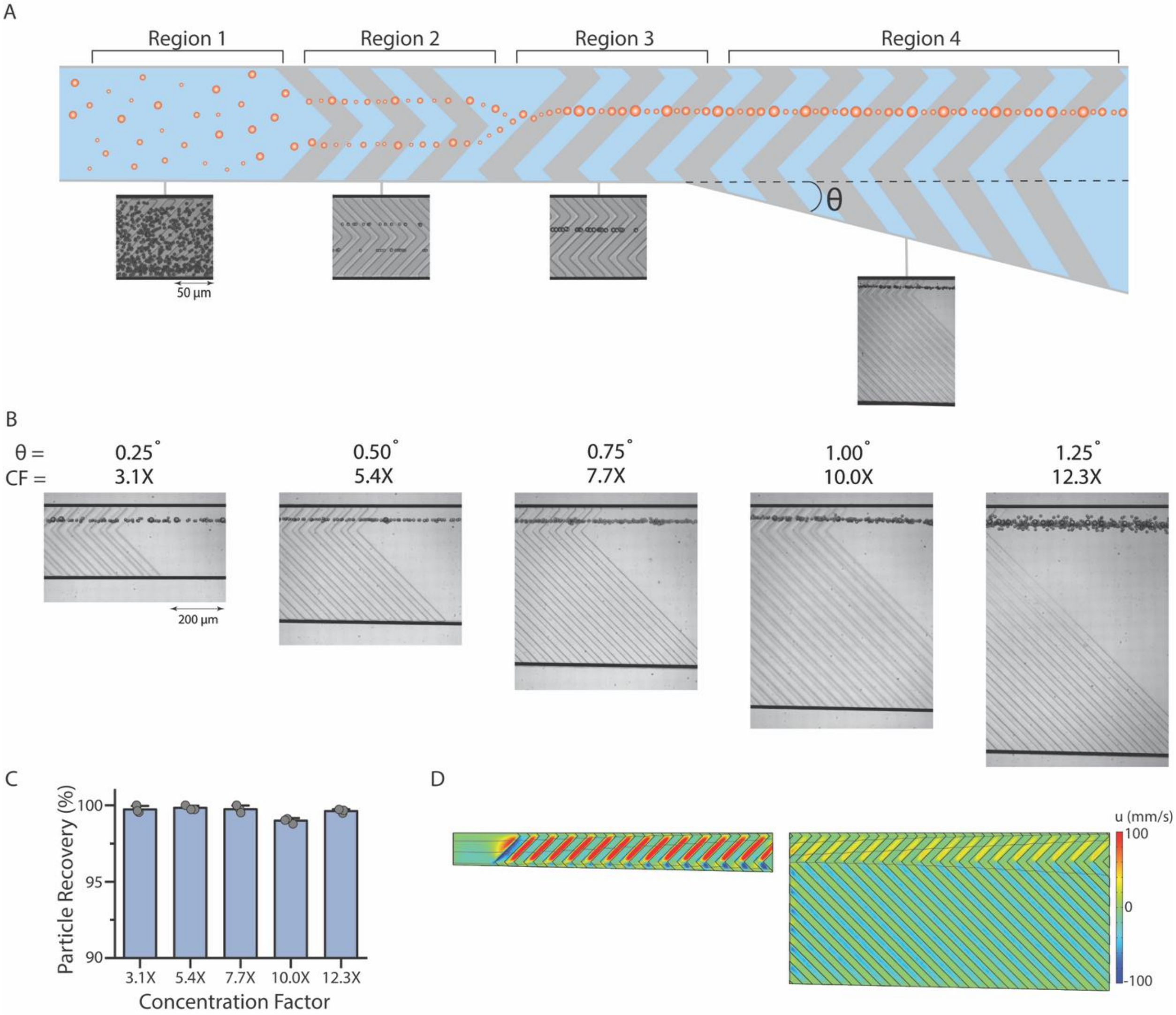

Figure S7. A RAMP device is used to successively align and concentrate polydisperse particles. (**A**) High-speed streak imaging showing the organization of polydisperse particles in various regions. (**B**) High-speed microscopy imaging shows a polydisperse mixture of particles (10 and 20 μm diameters) being concentrated with concentration factors (CFs) of 3.1X, 5.4X, 7.7X, 10.0X, and 12.3X. (**C**) RAMP cell concentration results in recovery greater than 99% for each CF tested (n = 3 replicates per concentration factor). High-speed videos show the polydisperse particle mixture flowing through concentration factors of 3.1X and 12.3X (**Movie S4** and **Movie S5**). (**D**) Flow simulation results show a change in the transverse velocity profile as fluid siphoning is implemented. Gray lines show streamlines.

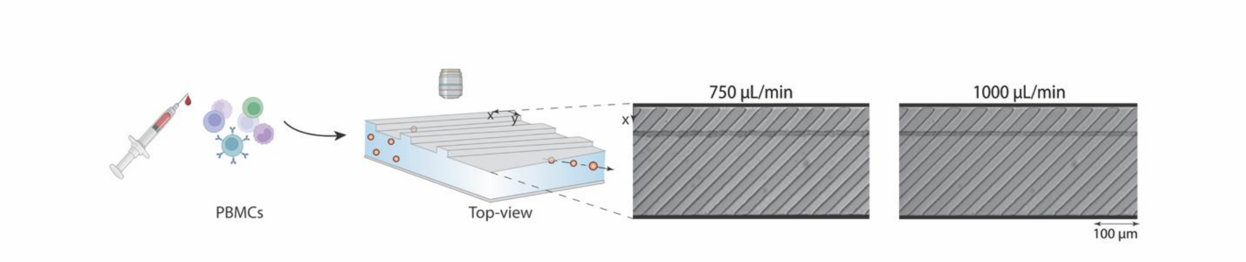

Figure S8. RAMP focuses peripheral blood mononuclear cells (PBMCs), showing its utility for clinically relevant cell suspensions.

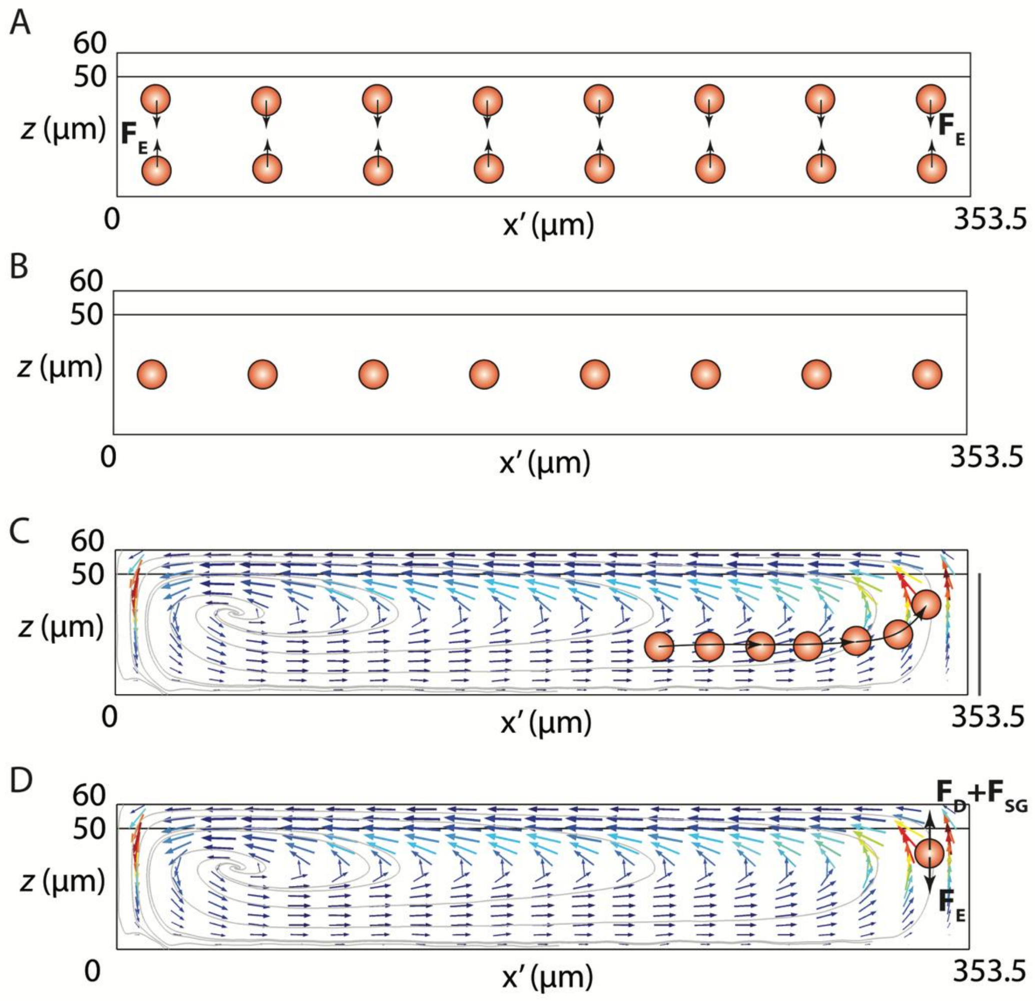

Figure S9. Schematics of viscoelastic focusing. (**A-B**) For the conditions tested in this work, elastic forces (F_E) dominate inertial forces; as a result, particles experience an elastic force towards the center of the rectangular channel. (**C-D**) Secondary flow produced by ridges sweeps particles towards the distant side wall of the channel, where elastic force towards the center of the channel balances the drag force due to vortex (F_D) and shear-gradient inertial lift force (F_{SG}).

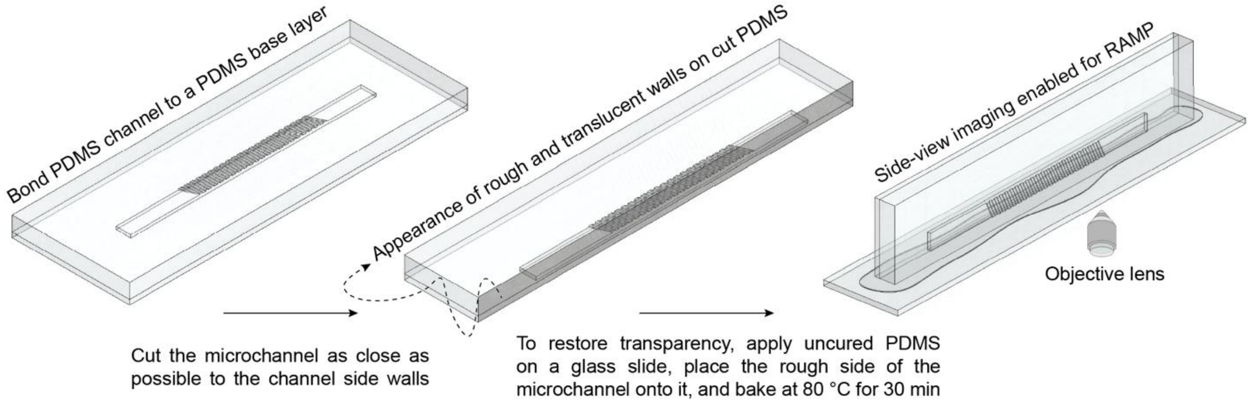

Figure S10. Fabrication process for PDMS device with a side-view (*y*-*z* plane) window.

Table S1. An overview of microchannel geometries used for inertial focusing with corresponding flow conditions, applicable particle size ranges, and focusing behavior.

Channel Type	Channel Visual	Tunable focusing	Size independent focusing	Flow Rate independent focusing	Focusing in height (Z direction)
Current Study (RAMP)	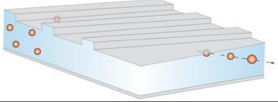	Yes	Yes	Yes	Single line
Serpentine and curvilinear	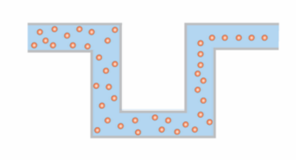	No	No	No	Double line
Obstacled curvilinear (*3–7*)	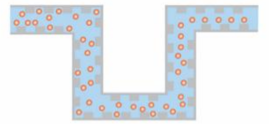	No	No	No	Double line
Asymmetric serpentine (*8*)	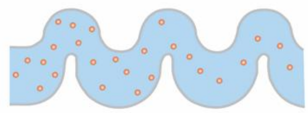	No	No	No	Double line
Spiral (*9–12*)	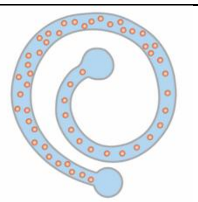	No	No	No	Double line
Obstacled-spiral (*13–15*)	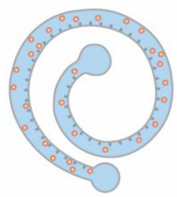	No	Yes	Yes	Double line
Contraction-expansion (*16–18*)	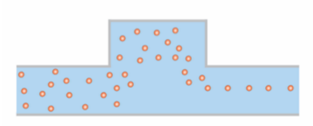	No	No	No	Double line
Reverse wavy (*19*)	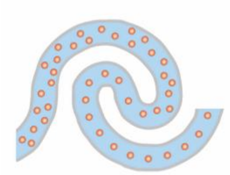	No	No	No	Double
Straight microchannel with rectangular cross-section (*20*, *21*)	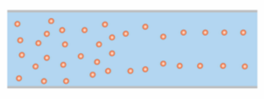	No	No	No	Double or multiple

Straight microchannel with triangular cross-section (*22–24*)	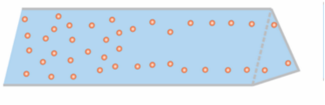	No	Yes (in some cases)	Yes (in some cases)	Single or double
Mixed channel cross-section in a straight channel (*25*)	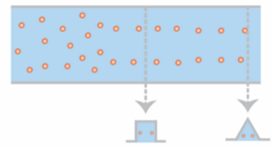	Yes (to some extent)	No	No	Single
Stepped straight microchannel (*26*)	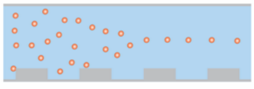	No	Yes	Yes	Single
Grooved microchannels (*27–29*)	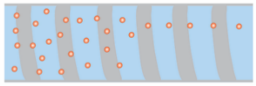	No	Yes	Yes	Single

Table S2. 10, 15, 20, and 30 μm particles' focus positions and standard deviations at flow rates of 250 to 1000 μL/min captured using high-speed microscopy imaging.

**Particle Size (μm)**	**Focus Position (μm)**			
	**250 μL/min Re = 25.2**	**500 μL/min Re = 50.4**	**750 μL/min Re = 75.6**	**1000 μL/min Re = 100.8**
10	57.8 ± 0.7	55.0 ± 0.6	52.2 ± 0.5	54.0 ± 0.5
15	54.2 ± 1.3	51.1 ± 0.9	51.3 ± 5.2	51.3 ± 2.8
20	54.4 ± 0.9	54.4 ± 1.3	56.2 ± 0.9	54.0 ± 3.2
30	59.5 ± 2.3	56.5 ± 1.8	53.8 ± 1.8	53.1 ± 2.6

Table S3. Focusing positions of 10 μm particle in *z*-direction at flow rates ranging from 250 – 1000 μL/min captured using fluorescence microscopy in *y*-*z* plane (side-view).

**Flow Rate (μL/min)**	**Mean Peak Position (μm)**
250	30.6 ± 0.6
500	31.5 ± 0.6
750	31.5 ± 0.6
1000	32.4 ± 0.5

Table S4. Focusing positions of 10 µm particles for small and large focus position shifts at a flow rate of 500 µL/min and *Re* of 50.4 captured using a high-speed camera.

**Ridge Position (µm)**	**Focus Position (µm)**	
	**10 µm Particles**	**10 and 15 µm Particles**
15	50.8 ± 0.7	
30	67.9 ± 0.6	
60	97.5 ± 1.0	
80	109.1 ± 0.6	109.0 ± 0.6
85	113.9 ± 0.5	114.7 ± 1.1
90	119.8 ± 0.6	121.1 ± 0.6
95	126.7 ± 0.6	

Table S5. Average cell and particle recovery in RAMP concentrators (n=3).

Concentration Factor	Cell Recovery (%)	Particle Recovery (%)
3.1X	97.6 ± 1.1	99.7 ± 0.2
5.4X	99.5 ± 0.2	99.8 ± 0.1
7.7X	98.4 ± 0.2	99.7 ± 0.2
10.0X	95.1 ± 0.5	99.0 ± 0.1
12.3X	96.8 ± 0.8	99.6 ± 0.1

Table S6. Deborah's number (*De*), Reynold's number (*Re*), and the Elasticity number (*El*) calculations for flow rates of 50 and 100 μL/min.

Flow Rate (μL/min)	*De*	*Re*	*El*
50	130.0	1.111	117.0
100	260.0	2.222	117.0

Movie S1.
Top-view of 10 μm particles flowing at 500 μL/min through a channel with inclined straight ridges.

Movie S2.
Top-view of 10 (left) and 30 (right) μm particles flowing at 500 μL/min through a channel with inclined straight ridges.

Movie S3.
Top-view of 10 μm particles flowing at 500 μL/min through a channel with center ridge structures at 80 μm from the channel side wall (left) and 90 μm from the channel side wall (right).

Movie S4.
Top-view of 9, 10, and 20 μm particles flowing through a RAMP concentrator with a concentration factor of 3.1X and operating pressure of 20 psi.

Movie S5.
Top-view of 9, 10, and 20 μm particles flowing through a RAMP concentrator with a concentration factor of 12.3X and operating pressure of 20 psi.

Movie S6.
Top-view of white blood and circulating tumor cells flowing through a RAMP concentrator with a concentration factor of 3.1X and operating pressure of 15 psi.

Movie S7.
Top-view of white blood and circulating tumor cells flowing through a RAMP concentrator with a concentration factor of 12.1X and operating pressure of 15 psi.

Movie S8.
Top-view of 20 μm particles flowing at 250 μL/min in whole blood through a device with RAMP.

References

1. A. D. Stroock, S. K. Dertinger, G. M. Whitesides, A. Ajdari, Patterning Flows Using Grooved Surfaces. *Anal. Chem.* **74**, 5306–5312 (2002).

2. D. Di Carlo, J. F. Edd, K. J. Humphry, H. A. Stone, M. Toner, Particle Segregation and Dynamics in Confined Flows. *Phys. Rev. Lett.* **102** (2009).

3. H. Cha, H. Fallahi, Y. Dai, S. Yadav, S. Hettiarachchi, A. McNamee, H. An, N. Xiang, N.-T. Nguyen, J. Zhang, Tuning particle inertial separation in sinusoidal channels by embedding periodic obstacle microstructures. *Lab. Chip* **22**, 2789–2800 (2022).

4. H. Cha, H. A. Amiri, S. Moshafi, A. Karimi, A. Nikkhah, X. Chen, H. T. Ta, N.-T. Nguyen, J. Zhang, Effects of obstacles on inertial focusing and separation in sinusoidal channels: An experimental and numerical study. *Chem. Eng. Sci.* **276**, 118826 (2023).

5. Y. Liu, J. Zhang, X. Peng, S. Yan, Deciphering the Evolution of Inertial Migration in Serpentine Channels. *Anal. Chem.* **96**, 14306–14314 (2024).

6. J. Zhang, S. Yan, R. Sluyter, W. Li, G. Alici, N.-T. Nguyen, Inertial particle separation by differential equilibrium positions in a symmetrical serpentine micro-channel. *Sci. Rep.* **4** (2014).

7. A. Özbey, M. Karimzadehkhouei, S. Akgönül, D. Gozuacik, A. Koşar, Inertial Focusing of Microparticles in Curvilinear Microchannels. *Sci. Rep.* **6**, 38809 (2016).

8. D. Di Carlo, D. Irimia, R. G. Tompkins, M. Toner, Continuous inertial focusing, ordering, and separation of particles in microchannels. *Proc. Natl. Acad. Sci.* **104**, 18892–18897 (2007).

9. M. E. Warkiani, G. Guan, K. B. Luan, W. C. Lee, A. A. S. Bhagat, P. Kant Chaudhuri, D. S.-W. Tan, W. T. Lim, S. C. Lee, P. C. Y. Chen, C. T. Lim, J. Han, Slanted spiral microfluidics for the ultra-fast, label-free isolation of circulating tumor cells. *Lab Chip* **14**, 128–137 (2014).

10. S. S. Kuntaegowdanahalli, A. A. S. Bhagat, G. Kumar, I. Papautsky, Inertial microfluidics for continuous particle separation in spiral microchannels. *Lab. Chip* **9**, 2973 (2009).

11. N. Herrmann, P. Neubauer, M. Birkholz, Spiral microfluidic devices for cell separation and sorting in bioprocesses. *Biomicrofluidics* **13** (2019).

12. A. A. S. Bhagat, S. S. Kuntaegowdanahalli, I. Papautsky, Continuous particle separation in spiral microchannels using dean flows and differential migration. *Lab. Chip* **8**, 1906 (2008).

13. J. Guo, S. Shen, N. Zhang, F. Zhang, Y. Niu, Y. Wu, Secondary flow enhancement in ultra-low aspect ratio microchannels with ordered Micro-obstacles for high-throughput cell focusing. *Microchem. J.* **216**, 114570 (2025).

14. S. Shen, Y. Zhang, K. Yang, H. Chan, W. Li, X. Li, C. Tian, Y. Niu, Flow-Rate-Insensitive Plasma Extraction by the Stabilization and Acceleration of Secondary Flow in the Ultralow Aspect Ratio Spiral Channel. *Anal. Chem.* **95**, 18278–18286 (2023).

15. L. Zhao, M. Gao, Y. Niu, J. Wang, S. Shen, Flow-rate and particle-size insensitive inertial focusing in dimension-confined ultra-low aspect ratio spiral microchannel. *Sens. Actuators B Chem.* **369**, 132284 (2022).

16. G.-Y. Kim, J. Son, J.-I. Han, J.-K. Park, Inertial Microfluidics-Based Separation of Microalgae Using a Contraction–Expansion Array Microchannel. *Micromachines* **12**, 97 (2021).

17. J. Zhang, M. Li, W. H. Li, G. Alici, Inertial focusing in a straight channel with asymmetrical expansion–contraction cavity arrays using two secondary flows. *J. Micromechanics Microengineering* **23**, 085023 (2013).

18. M. G. Lee, S. Choi, J.-K. Park, Inertial separation in a contraction–expansion array microchannel. *J. Chromatogr. A* **1218**, 4138–4143 (2011).

19. Y. Zhou, Z. Ma, Y. Ai, Sheathless inertial cell focusing and sorting with serial reverse wavy channel structures. *Microsyst. Nanoeng.* **4**, 5 (2018).

20. J. Zhou, I. Papautsky, Fundamentals of inertial focusing in microchannels. *Lab. Chip* **13**, 1121 (2013).

21. C. Liu, G. Hu, X. Jiang, J. Sun, Inertial focusing of spherical particles in rectangular microchannels over a wide range of Reynolds numbers. *Lab. Chip* **15**, 1168–1177 (2015).

22. P. Mukherjee, X. Wang, J. Zhou, I. Papautsky, Single stream inertial focusing in low aspect-ratio triangular microchannels. *Lab. Chip* **19**, 147–157 (2019).

23. E. Cho, J. Kim, M. K. Aslan, Y. Meng, S. Stavrakis, A. deMello, Tunable viscoelastic size-based particle separation in straight microchannels with triangular cross-sections. *Sens. Actuators B Chem.* **414**, 135892 (2024).

24. J. Kim, J.-R. Lee, T.-J. Je, E. Jeon, W. Lee, Size-Dependent Inertial Focusing Position Shift and Particle Separations in Triangular Microchannels. *Anal. Chem.* **90**, 1827–1835 (2018).

25. J. Kim, J. Lee, C. Wu, S. Nam, D. Di Carlo, W. Lee, Inertial focusing in non-rectangular cross-section microchannels and manipulation of accessible focusing positions. *Lab. Chip* **16**, 992–1001 (2016).

26. A. J. Chung, D. R. Gossett, D. Di Carlo, Three Dimensional, Sheathless, and High-Throughput Microparticle Inertial Focusing Through Geometry-Induced Secondary Flows. *Small* **9**, 685–690 (2013).

27. Q. Zhao, D. Yuan, S. Yan, J. Zhang, H. Du, G. Alici, W. Li, Flow rate-insensitive microparticle separation and filtration using a microchannel with arc-shaped groove arrays. *Microfluid. Nanofluidics* **21**, 55 (2017).

28. Q. Zhao, J. Zhang, S. Yan, D. Yuan, H. Du, G. Alici, W. Li, High-throughput sheathless and three-dimensional microparticle focusing using a microchannel with arc-shaped groove arrays. *Sci. Rep.* **7**, 41153 (2017).

29. Q. Zhao, D. Yuan, S.-Y. Tang, G. Yun, S. Yan, J. Zhang, W. Li, Top sheath flow-assisted secondary flow particle manipulation in microchannels with the slanted groove structure. *Microfluid. Nanofluidics* **23** (2019).
